# A Novel Starch from *Talisia floresii Standl* Seeds: Characterization of Its Physicochemical, Structural and Thermal Properties

**DOI:** 10.3390/polym15010130

**Published:** 2022-12-28

**Authors:** Jorge C. Canto-Pinto, Eduardo Reyes-Pérez, Emilio Pérez-Pacheco, Carlos R. Ríos-Soberanis, Yasser A. Chim-Chi, José D. Lira-Maas, Raciel J. Estrada-León, Mario A. A. Dzul-Cervantes, José H. Mina-Hernández

**Affiliations:** 1 Tecnológico Nacional de México, Campus Instituto Tecnológico Superior de Calkiní, Cuerpo Académico Bioprocesos, Av. Ah-Canul, Calkiní C.P. 24900, Campeche, Mexico; 2 Centro de Investigación Científica de Yucatán, A.C., Unidad de Materiales, Calle 43 No. 130 x 32 y 34, Colonia Chuburná de Hidalgo, Mérida C.P. 97205, Yucatán, Mexico; 3 Group Materiales Compuestos, Universidad del Valle, Calle 13 No. 100-00, Cali 76001, Colombia

**Keywords:** *Talisia floresii* seed, starch, physicochemical properties, functional properties, food package

## Abstract

Colok seed (*Talisia floresii Standl*) represents 80% of the total fruit weight and is obtained from trees that grow mainly in Yucatan Peninsula. The aim of this work was the physicochemical characterization from colok starch seeds as an alternative to conventional sources and to identify its characteristics for potential applications in different industrial sectors. Starch yield was 42.1% with low levels of lipids, ashes and fibers. The amylose content was 33.6 ± 1.15%. The gelatinization temperature was 85 ± 0.25 °C. Color analysis resulted in a starch with an intermediate luminosity, reflecting a dark color. Finally, in morphology, starch granule exhibited an average size of 18.7 μm, spherical, uniform and without fractures. Overall results demonstrated that isolated colok starch can be used in food products that require high processing temperatures, such as sauces, cookies, noodles, bread and food packages.

## 1. Introduction

Nowadays, the industry has extended the use of a great diversity of materials for the production, packing, conservation and commercialization of food. Many efforts are oriented towards research and progress in food packaging, for non-polluting and biodegradable sources. Research points out starch as a material of natural origin that is used in different industrial sectors, for example, drugs, adhesives, food, and food packaging. Starch is the most popular plant polysaccharide due to its abundance, cost-effectiveness, and excellent film-forming capabilities. It is stored as semi-crystalline granules in cereals, grains, roots, tubers, leaves, seeds, fruits and pollen. It is composed of two different polysaccharides: amylose, which possesses a linear structure formed from the union of D-glucose through α 1–4 bonds, and amylopectin, where D-glucose, in addition to being joined by α 1–4 bonds, exhibits branches linked by α 1–6 bonds [1]. Recently, the study of starch from non-conventional sources has been increased to broaden the variety of industrial use. The starches from green plantain (*Musa paradisiaca*), mafafa (*Xanthosoma robustum*), ramon (*Brosimum alicastrum*) and bitter vetch (*Vicia ervilia*) have shown different physicochemical, structural and functional properties [2,3,4,5]. The food industry is searching for new sources of macromolecules with extensive physicochemical and functional properties that can be used as additives. Particularly, starches that possess viscosity and allow the formation of transparent gels are required [6]. Rice, wheat and corn fulfill these characteristics sufficiently [7,8,9]. However, it is necessary to emphasize that these serve as foods in different cultures, so it is necessary to investigate new sources of starches. Nonetheless, new sources, mainly from endemic plants, exist that have not yet been studied, such as the seeds of colok (*Talisia floresii Standl*).

*T. floressi Stand* is a tree that can reach up to 18 m in height, also known as colok in Mexico, which is distributed in the southeast, particularly in Campeche, Yucatán, Tabasco and Quintana Roo [10,11]. The fruit is rigid, subglobose, 5 cm long and is made up of three layers: the velvety yellowish-green peel, a white aril that is edible, and the seed, which constitutes 80% of the fruit. The fruits are collected from the September–November season [12]. The seed contains in its interior a large endosperm rich in starch that can be used in different applications, such as in the food industry. The use of a non-conventional source of starch can increase the desired properties of value-added food products. For this reason, the aim of this study is to characterize the physical–chemical, structural, thermal and functional properties of starch isolated from colok seed flour. The information obtained will support in defining the effective applications of colok seed flour starch in food and food package industry. Actually, this seed is rarely used for human consumption among the population of the Yucatan Peninsula. The use of a non-conventional source of starch can increase the desired properties of value-added food products compared with cereals, such as corn or rice. For this reason, the aim of this study is to characterize the physical–chemical, morphological, thermal and functional properties of a new, non-conventional source of starch isolated from colok seed. The information obtained will support in defining the effective applications of colok starch in food and the food packaging industry.

## 2. Materials and Methods

### 2.1. Materials

**Figure 1:**
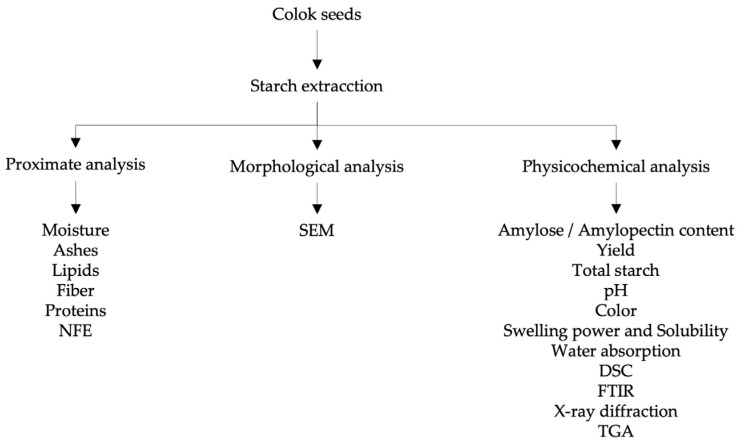
Schematic diagram for the isolation and characterization of *Talisia floresii Standl*.

Colok fruits were collected in the municipality of Calkiní, Campeche, México, during September 2020. Fruits were selected avoiding overripe samples, with imperfections or damage. In order to obtain seeds, colok fruit was pulped manually. The seeds were dried in a convection oven (Shell Lab 1350FX-10) for 72 h at 40 °C. Finally, the endosperm was pulverized in a commercial blender (Osterizer^®^) at intervals of 10 s to be posteriorly sifted through a 100 mesh to obtain the flour. The flour obtained was stored in hermetically sealed glass containers until its use. Figure 1 shows a schematic diagram of research.

### 2.2. Native Starch (NS) Extraction

**Figure 2:**
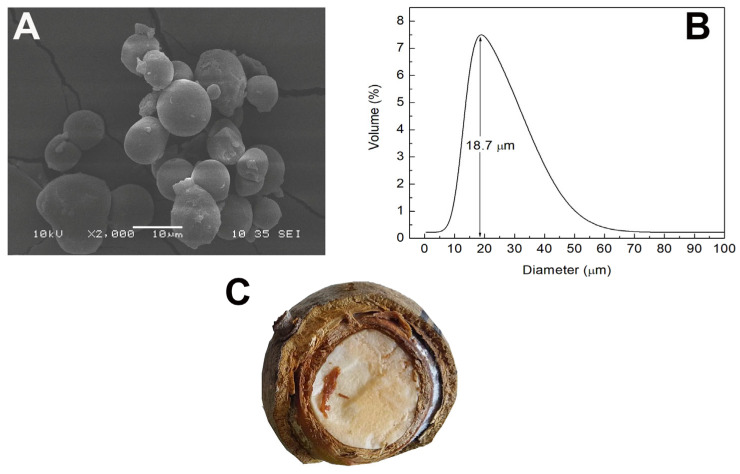
(**A**) Micrograph on colok starch. (**B**) Average size of colok granule. (**C**) Colok seed.

Native starch was obtained by alkaline hydrolysis of colok flour seed (see Figure 2C), following the procedure described by Estrada-León et al. [13] with modifications. Then, 500 g of flour were dissolved in a sodium bisulfite (Sigma-Aldrich, St. Louis, MO, USA, 243973) solution at 0.1% and 5 L of water and left to stand for 12 h. Subsequently, pH was adjusted to 10 using an NaOH solution 1 N (Sigma-Aldrich, St. Louis, MO, USA, S5881), leaving it to stand for 30 min to settle; then, the suspension was filtered through a No. 100 sieve to remove the fiber, followed by centrifugation at 3000 rpm for 15 min. Finally, the supernatant was removed, and the sediment was dried in a convection oven for 24 h at 45 °C. The dried starch was ground in an IKA MF-10 mill and passed through a No. 100 sieve [14,15].

### 2.3. Chemical Proximate Analysis

The proximal composition of the obtained starch was carried out according to the AOAC methods [16,17] for humidity (925.10), ashes (923.03), proteins (920.87) and lipids (920.39). Crude fiber was determined by acid and alkaline digestion as reported in the literature [18,19]. Carbohydrates were estimated as Free Nitrogen Extract (ELN), that is, by difference against 100%. Additionally, pH was determined with a Metrohm 827 potentiometer in a 1% (*w*/*v*) dispersion at room temperature.

### 2.4. Color Determination

Native starch color was measured in triplicate using a Hunter Lab MiniScan EZ colorimeter. Parameters were estimated in the CIELAB space as L* (lightness, from 0 = black to 100 = white), a* (+a = redness, −a = greenness) and b* (+b = yellowness, −b = blueness) and the final result was expressed as hue angle (h) with Equation (1) and chromaticity C* (intensity of the tone) was calculated with Equation (2) [20,21]:

(1) $$h=\tan^{-1}\frac{b*}{a*}$$

(2) $$C*=\sqrt{{\left(a*\right)}^{2}+{\left(b*\right)}^{2}}$$

### 2.5. Apparent Amylose Content

Amylose content was determined according to Ratnayake et al. [22]. This analysis consisted of solubilizing starch in dimethyl sulfoxide after being exposed to an iodinated solution. Amylose content was determined with a standard curve, using different concentrations of potato amylose (ranging from 0 to 100% amylose), expressed as a percentage. Amylopectin content was calculated by difference by subtracting amylose content, using the colorimetric method proposed by Morrison and Laignelet [23,24].

### 2.6. Total Starch

Total starch was determined using the starch assay kit (Sigma-Aldrich, STA20), which is based on the hydrolysis of starch to glucose catalyzed by α-amylase and amyloglucosidase. Glucose is oxidized to gluconic acid and hydrogen peroxide by glucose oxidase. Hydrogen peroxide reacts with ο-dianisidine in the presence of peroxidase to form a colored product. Oxidized ο-dianisidine reacts with sulfuric acid to form a stable colored product. The intensity of the pink color measured at 540 nm is proportional to the original glucose concentration. The analyses were performed according to the instructions supplied with the kits [25].

### 2.7. Differential Scanning Calorimetry (DSC)

Starch gelatinization was measured using a DSC-6 (Perkin Elmer Corp., Norwalk, CT, USA) according to Estrada et al. [13]. Approximately, 1 mg of starch was weighed into an aluminum sample pan. Next, 3 μL of water was added with a microsyringe to obtain a starch:water ratio of 1:3 (*w*/*w*) in the DSC pans, which were sealed and left at room temperature for 1 h. Pans were heated from 25 to 110 °C (temperature increased at a rate of 10 °C/min). The sample chamber was flushed with nitrogen to avoid moisture condensation. An empty aluminum pan was used as the reference. The onset (T_o_), peak (T_p_) and conclusion temperatures (T_c_) were recorded. The enthalpy change of the thermal transition (ΔH_gel_) was estimated by integrating the area between the thermogram and a base line under the peak and was expressed as J/g dry weight of starch [26].

### 2.8. Thermogravimetry Analysis (TGA)

Thermal properties of native starch (NS) were measured with a TGA Perkin Elmer 7/DX thermal analyzer. Next, 6 mg of starch was placed in a platinum pan and heated from 50 to 500 °C (rate 10 °C/min) to observe the temperature at which decomposition occurred. During the entire process, nitrogen at 3.7 bar was delivered through the system containing the sample at 20 mL/min [27].

### 2.9. Fourier Transform Infrared (FTIR) Analysis

The FTIR spectroscopy analyses were performed on NS in order to characterize qualitatively the organic compounds of the solids by using the transmission technique. Samples were prepared by grinding 15 mg of solid powder starch with 130 mg potassium bromide (KBr) powder and then pressing the mixture into a tablet. The total mass of the solid powder was 150 mg and were recorded for 5 specimens of 30 mg of mass. The FTIR spectrum of the powder complexes was measured at room temperature with a ThermoNicolet (Nexus 670-FTIR) spectrometer in a spectral range of 4000 and 400 cm^−^^1^ [28].

### 2.10. X-ray Diffraction (XRD)

In order to analyze the X-ray diffraction, a Siemens diffractometer, model D-5000, operating at Cu-Kα radiation wavelength (λ = 1.54 Å), 40 kV, 30 mA and sampling interval of 0.02° was used. Scattered radiation was detected in the angular range of 5–35° (2θ).

### 2.11. Scanning Electron Microscopy (SEM) and Particle Size

The morphological characteristics of NS were observed using an SEM. Starch samples were mounted on a metallic slide and the examination was performed with a scanning electron microscopy JEOL JSM 6360 LV electron probe microanalyzer at 15 kV in low vacuum. Subsequently, the starch was suspended in an appropriate volume of distilled water and placed in a Beckman Coulter LS100Q laser diffraction particle size analyzer with a precision of ±1% [29].

### 2.12. Swelling Power and Solubility

The procedure of Rafiq et al. [30] was used for determination of swelling power and solubility of starch. Solubility and swelling power patterns at 60, 70, 80 and 90 °C were determined. Starch slurry (1 g/100 mL, starch d.b.) in centrifuge tubes was heated at 60, 70, 80 and 90 °C for 30 min. The tubes, after cooling, were centrifuged at 2500 rpm during 15 min, the supernatant was decanted in petri plates, evaporated, and dried at 105 °C for 5 h until constant weight was achieved and were weighed to calculate the solubility. The residue was weighed for swelling power estimation. Swelling power and solubility were calculated with Equations (3) and (4):

(3) $$\mathrm{WSI}=\frac{\mathrm{weight}\;\mathrm{of}\;\mathrm{solid}\;\mathrm{solubles}\;\left(g\right)}{\mathrm{weight}\;\mathrm{of}\;\mathrm{sample}\;\left(g\right)}\times 100\%$$

(4) $$\mathrm{swelling}\;\mathrm{power}\;\left(g/g\right)=\frac{\mathrm{weight}\;\mathrm{of}\;\mathrm{gel}\;\left(g\right)}{\mathrm{weight}\;\mathrm{of}\;\mathrm{sample}\;\left(g\right)-\mathrm{weight}\;\mathrm{of}\;\mathrm{solid}\;\mathrm{soluble}\;\left(g\right)}$$

### 2.13. Water Absorption (WAI)

The water absorption capacity was determined according to Delatte et al., and Torbica, Belović and Tomić [31,32] with modifications. A total of 40 mL of a 1% (d.b.) starch suspension was prepared in distilled water at 30 °C. The suspension was heated at a rate of 1.5 °C/min until it reached 60, 70, 80 or 90 °C and was kept at these temperatures for 30 min with constant stirring. It was allowed to cool to room temperature and centrifuged at 2500 rpm for 15 min, in a GS-15R centrifuge (Beckman Instruments, Inc. Brea, CA, USA). The resulting gel was weighed. The water absorption capacity for each temperature was calculated as the weight (g) of the gel per g of dry sample. The WAI was calculated and expressed as in Equation (5):

(5) $$\mathrm{WAI}=\frac{\mathrm{Weight}\;\mathrm{of}\;\mathrm{sedminet}\;\left(g\right)}{\mathrm{Weight}\;\mathrm{of}\;\mathrm{sample}\;\left(g\right)}\times 100\%$$

### 2.14. Statistical Analysis

The quantitative variables of the proximal analyses and the physicochemical characteristics of the colok starch were described by estimating their mean and standard deviation (descriptive statistics). All determinations were performed in triplicate.

## 3. Results and Discussion

### 3.1. Chemical Proximate Analysis

At harvest time, the fruit presented an average weight of 44 ± 0.80 g and an average diameter of 44.2 ± 0.5 mm. The starch yield obtained from colok seeds was 42.1%, expressed on a dry basis. This value is slightly higher than that reported by some authors for other types of starch. Corn starch yield, extracted under conditions similar to those described in this work, was 40% [33]. These differences may be due to the nature of the plants, since corn is a cereal, while colok is classified as a *sapindaceae* [12]. Regarding the proximal analysis, the obtained values are reported in Table 1. Colok starch contains 9.49 ± 0.52% moisture, which is within the range that is considered acceptable for dry powdered products (<15%) and exhibited a moisture content similar to that reported for parota seed starch (*Enterolobium cyclocarpum*) [13] under the same extraction conditions already described. Furthermore, colok starch had a lower moisture percentage than recommended (<20%) for other starches derived from typical sources, such as potatoes. The amylose and amylopectin content found in this work is similar to others, such as corn and potato. This content varies from the source of starch. In this sense, the relationship between these is too important. This arrangement is related to the starch gelatinization, and, when starch is heated, the viscosity is also increased. In addition, high amylose content could be considered as a smart reserve for use as an obstruction in packaging materials [34].

Ash content is a parameter linked to the mineral content. In starches, for example, phosphorus is a mineral that exerts a significant influence as it is responsible for the swelling and stability of the paste. Compared to some cereals, such as corn grain [35], and tubers, such as potatoes [36], with ash content values of 0.58 and 37%, respectively, the colok presented a higher value; consequently, high rates of swelling and paste stability are expected. The fat content found in colok starch was much higher than that reported for parota starch that contained only 1.2% [13]. However, these authors mention that the botanical source from which the starch is extracted is essential. Colok fiber content was found to be around 3.62%; similar values, between 3.6 and 4.84%, were obtained in starches extracted from quinoa (*Chenopodium quinoa Willd*) [37].

### 3.2. Morphology of Starch Granule

Scanning electron microscopy (SEM) analysis of starch isolated from colok fruit seeds is shown in Figure 2A. Starch granules’ functional properties are significantly influenced by their granule size and size distribution. Through this analysis, shape of the granules of this non-conventional native starch was identified. The shape of these granules is spherical, uniform and without fractures. Therefore, it indicates that the alkaline extraction of this macromolecule does not affect the integrity of the granules, nor modify their shape, although morphological traits can vary depending on the cultivar, plant growth, environmental circumstances, and techniques of extraction and purification; smooth surfaces without fractures suggest purity in starch extraction. Granule size distribution is shown in Figure 2B. Regarding its size, the average report is 18.7 μm; hence, agreeing to the classification appointed by Lindeboom et al. [38], and according to their size, these granules are classified as medium (between 10 and 25 μm). However, these particles are larger than those related to native corn starch, whose granules measure 15 μm [39]. Starch granule size plays an essential role that influences pasting parameters of starches. Applications that call for relatively small starch granules, such paper coating, can use starch with small starch granules.

### 3.3. Physicochemical Characteristics

Regarding the pH, colok starch presented a similar result to those obtained in previous investigations, for parota, a value of 6.44 ± 0.09 was observed despite the alkaline extraction method and for quinoa, a pH value of 6.45 (Table 2). High pH values are favorable for the ionization of polysaccharides that conform to starch structure [37,38,40]. The pH value denotes a suspension with a neutral pH.

The color analysis (Table 3) resulted in a starch with an intermediate luminosity, reflecting a dark color in comparison to parota seeds starch that presented greater whiteness, having values of luminosity L = 86.5 and color angle (hue) 92.3 [13]. Similarly, the a parameters indicate red tones, while the yellow tones given by the b parameter are greater. This coincides with the value of the hue angle, which indicates a tendency to red tones. Colok starch is appropriate for use in the bread industry. Lower height and specific volume in muffins are improved too [41]. The percentage of amylose in colok starch presented a value of 33.6%, similar to those reported for starches obtained from rice that can present values that oscillate around 37% [42] and from corn with values of 32% [43]. Consequently, the amylopectin value will be obtained by the difference at 100% [44]. It is important to state that the amylose/amylopectin ratio provides information on the functional properties of any starch, since amylose forms and provides stability to gels, while amylopectin confers viscosity. According to the results (amylose/amylopectin = 0.5 ± 0.01), this starch might be employed as a thickening agent in products that need a high level of viscosity because it has a low tendency to retrograde. For the above mentioned, colok starch is adequate for use in the bread industry. Lower height and specific volume in muffins are improved too.

### 3.4. Swelling Power (SP) and Solubility (IS)

**Figure 3:**
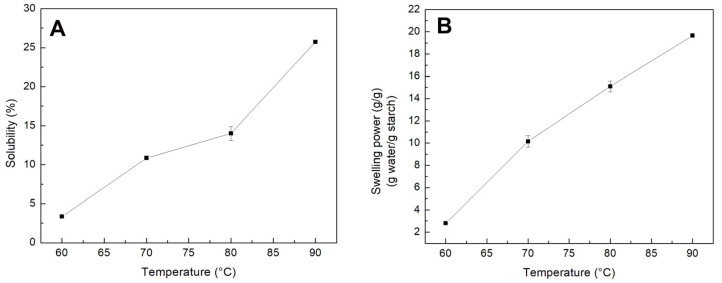
(**A**) Solubility. (**B**) Swelling power of isolated colok starch at different temperatures.

The swelling power and solubility represent evidence of interaction between the amorphous and crystalline areas. In addition, it is influenced by amylose and amylopectin characteristics [45]. Specifically, the SP of starch gives information on the mass of water that can be absorbed by one gram of starch granules in the presence of excess water at high temperature. Coincidentally, SP value also indicates the degree of crystallinity of the starch granules. Starch granules with lower crystallinity have a higher tendency to absorb more water and swell to a larger extent. Commonly, tuber starches have lower crystallinity and hence exhibit high SP due to their higher amylopectin content. Starch granules with greater SP usually show a harmonious higher amylose leaching [46]. Figure 3B shows the result of the swelling power analysis as a function of temperature. In this Figure, an increment in the swelling power as the temperature increased at a constant change rate was observed. This behavior may be due to the fact that, at high temperatures, there is a progressive relaxation of the binding forces within the granule, which implies an increase in SP while augmenting temperature. Likewise, it is observed that at 60 °C the swelling power of the starch was minimal; however, as the temperature increased to 90 °C, the SP increased to a maximum of 19.6 g water/g starch, which represents an 85% increase from 60 to 90 °C. This behavior was similar to that reported for other starches [47]. Solubility of starch is the result of the leaching of amylose when the starch is heated under an excess amount of water. As the starch granules imbibe more water and swell, the amylose dissociates from and diffuses out of them. Figure 3A shows the result of the solubility analysis performed on colok starch. As can be noticed, there is an increase in IS at a constant change rate in the temperature range from 60 to 70 °C. Likewise, a decrease in IS was observed at a lower rate of change in the temperature range of 70 to 80 °C. Finally, a new increase in IS was observed at a higher change rate. Moreover, at 60 °C, IS is low and increases by 87% when going from 60 to 90 °C. According to Mbougueng et al. [48], the higher water absorption capacity could be due to the size of the starch granule. In addition, Betancur et al. [49] mention that the increase in this water absorption capacity would be due to the presence of hydrophilic groups that retain water. Kaur et al. [50] found differences in swelling power for small (30.4–30.7 g water/g starch) and large (25.7–27.2 g water/g starch) granule fractions of starches from different potato cultivars harvested in India. Furthermore, Lin et al. [51] mention that the higher solubility of starch can be attributed to a higher solubilization of polymers from starch granules possessing weaker rigidity, when heated at high temperature.

### 3.5. Water Absorption

**Figure 4:**
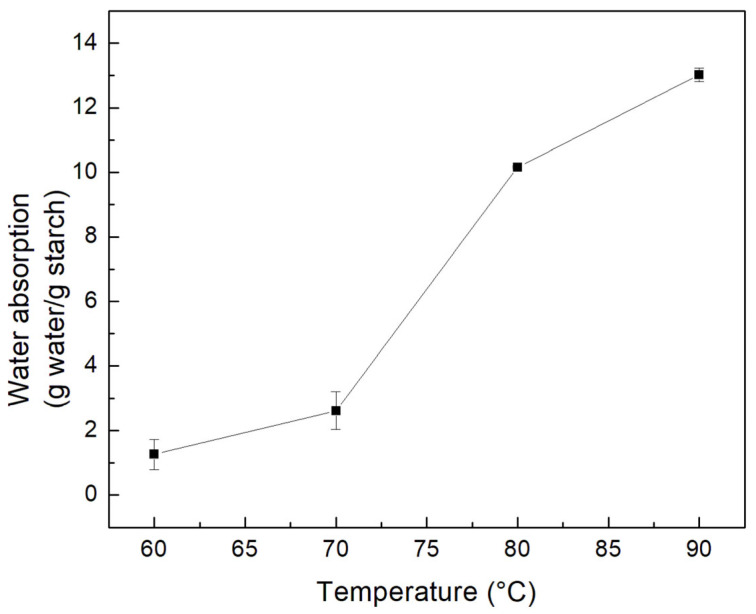
Water absorption of colok starch at different temperatures.

Figure 4 presents the behavior of water absorption in relation to g of water and g of starch. Just as in the case of solubility and swelling power, an increase is observed as the temperature rises. However, it is noteworthy that the greatest increase occurs from 70 °C, where the water absorption increases by 8 g. The increment continues, but slowly, until reaching 14 g at 90 °C. This behavior of increase with respect to temperature is similar to that reported in the literature [52]. Colok starch resists swelling between 60 and 70 °C, according to the values given for water absorption at various temperatures. This is understandable given the high gelatinization temperature observed (85 ± 0.25 °C). Therefore, its use as a food additive in the preparation of sauces in the food industry is suggested.

### 3.6. Diferential Scanning Calorimetry Analysis (DSC)

DSC technique measures the amount of heat involved in the gelatinization of starch. This property is related to various factors, such as the size, proportion and type of crystalline organization and the ultrastructure of the starch granules. Moreover, as mentioned before, starch is composed of two types of macromolecules: amylose and amylopectin. The first is related to the amorphous structure of the starch and the second to the crystalline fragment. Initially, gelatinization occurs in the amorphous part because hydrogen bonds rapidly weaken in such areas. However, amylopectin plays an important role in crystallinity. A higher content of amylopectin increases structural stability, resistance to gelatinization and the energy that initiates gelatinization, which leads to a rise in the transition temperature and enthalpy of gelatinization (ΔH_gel_) [53]. DSC was used to study starch gelatinization involving the disruption of the native colok starch structure. Gelatinization temperatures (T_o_, T_p_ and T_c_) and enthalpy of colok starch are shown in Table 4.

Gelatinization process begins at a temperature of 81 ± 0.5 °C (T_o_) where the beginning of the swelling of the granule due to the entry of water is identified, presumably in the amorphous structure that is related to the degree of crystallinity. When temperature is risen, the starch granules undergo fragmentation until the amylose (amorphous structure) is completely solubilized while the crystalline zone of starch remains in an aqueous solution. Under conditions of excess water, hydrogen bonds in the amorphous region of the granule are broken, allowing water to associate with the free hydroxyl groups. This is defined by the mobility of the polymer chains above the value of the glass transition temperature, causing the change from glassy to rubbery state. This change, in turn, facilitates molecular mobility in the amorphous regions, being a reversible process and allowing grain swelling. The granule expands as the polymers hydrate. Subsequently, an irreversible molecular transition occurs related to the dissociation of the double helices typical of the crystalline region. The highest value of heat absorption was observed at 85 ± 0.25 °C (T_p_) in which the starch passes into a rubbery state due to the rupture of its granules. This temperature is associated with the quality of the crystalline structure, the higher it is, the more cohesive and stable the crystalline region. The process culminates at a temperature of 90 ± 0.2 °C (T_c_), corresponding to the melting temperature of the strong crystal structure. High T_p_ values in colok starch mean that it requires more energy to produce starch gelatinization and may be influenced by its low swelling capacity, high amylose content, medium size, higher proportion of crystalline region by the interaction of starch chains and internal arrangement of starch fractions within the granule. The T_o_, T_p_, T_c_ values of colok starch were slightly higher than those reported by Sudheesh et al. [54] for Kithul starch and similar to those reported by Pérez-Pacheco et al. for ramon starch. The gelatinization enthalpy (ΔH_gel_) is indicative of the loss of molecular order (crystalline region) which occurs in the starch granules during gelatinization. The value of ΔH_gel_ calculated for colok starch is lower than the value reported for ramon starch [3] and similar to that reported for common buckwheat starch [55]. The high value of ΔH indicates that colok starch exhibits a well-organized microstructure and therefore requires more energy to gelatinize.

### 3.7. Fourier Transform Infrared (FTIR) Analysis

**Figure 5:**
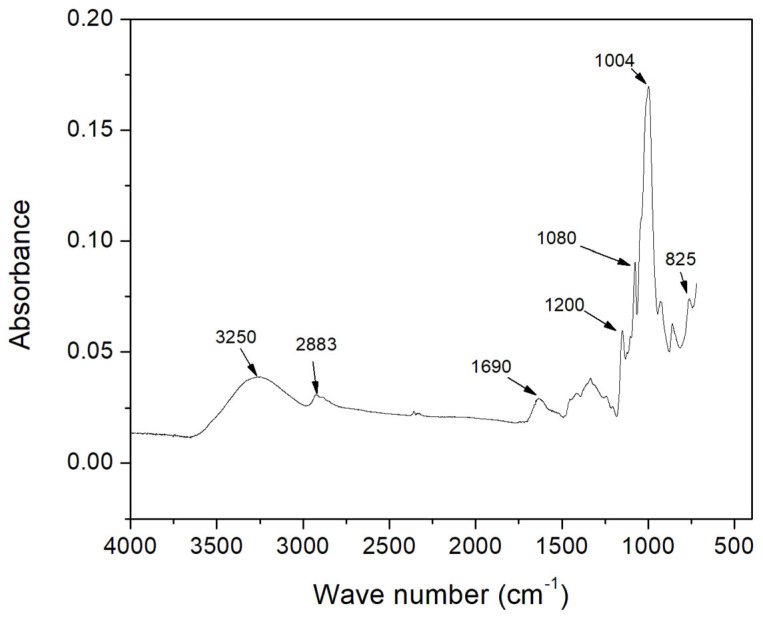
FTIR spectra of native colok starch.

In order to obtain information about the functional groups in the isolated colok starch, Fourier Transform Infrared (FTIR) spectroscopy analysis was performed. In Figure 5, the infrared spectrum for starch obtained from colok seeds can be observed. In the spectrum, an absorption band around 3270 cm^−^^1^ is shown, which can be attributed to the vibration of the hydroxyl groups [25]. The broad nature of this band could indicate that the starch exhibits strong hydrogen bonding interaction among themselves, and the water molecules present in it [56]. Some band peaks can also be observed around 2920 cm^−^^1^, these peaks could be attributed to the stretching vibrations of the bonds belonging to C-H groups [57]. Likewise, the absorbance band appearing around 1630 cm^−^^1^ can be correlated to the bending of O-H bonds belonging to the presence of water absorbed by the starch [58]. In the same way, “fingerprint” area displays the characteristic peaks of the starch; in this region, the highest intensity peaks can be appreciated at 1150 cm^−^^1^, 1080 cm^−^^1^ and 1004 cm^−^^1^. These peaks could be attributed to the vibrations of glucose C–O–C bonds. Likewise, the bands exhibited around 926 cm^−^^1^, 860 cm^−^^1^ and 764 cm^−^^1^ could correspond to the pyranose ring [59]. In starches, an amorphous and a crystalline region coexists; the amount of each one of them is important in order to predict the response of this polysaccharide when it is processed, and to identify its behavior when it is stored [13]. In this sense, several authors [13,59,60] have reported the relationship between the bands around 1047 cm^−^^1^ referred to the ordered region and the band around 1022 cm^−^^1^ referred to the amorphous region. The relationship between these bands represents the order in the starches [59], in the case of the starch obtained from the seeds of colok (*Talisia floresii Standl*) is 1.7. Other authors have reported values similar to this, such as the one obtained from ramon (*Brosimum alicastrum*) seeds, which report a value of 1.6 [59]. This value is even higher than that obtained for huaya (*Melicocus bijugatus*) with a value of 0.9 [25].

### 3.8. X-ray Diffraction

**Figure 6:**
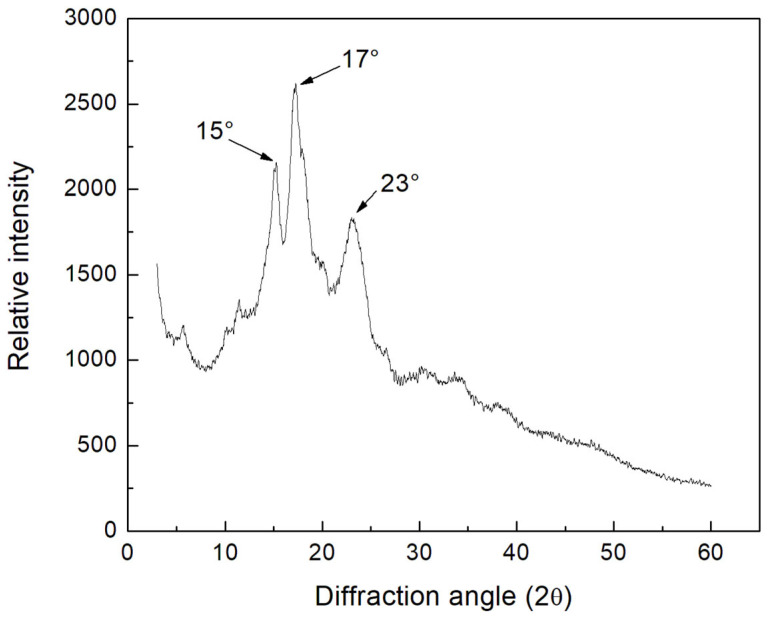
X-ray diffraction of native colok starch.

Figure 6 shows the results of the X-ray diffraction analysis. According to the water content and packing configuration of amylopectin, starch can present three main types of crystallinity, defined as A, B and C, distinguished by intensity X-ray diffraction lines [57]. Variations in the region between 6° < 2θ < 35° in X-ray diffraction patterns show that two different groups corresponding to type A and B starch structures are present [61]. Type A starches have two strong diffraction peaks around 2θ at about 15° and 23° and a doublet around 2θ at 17° and 18°, while type B starch possesses a strong diffraction peak around 2θ near 17°, as well as small peaks around 2θ near 15° and 24°. Diffractogram obtained for colok (*Talisia floresii standl*) starch is similar to that reported by other authors corresponding to ramon (*Brosimum alicastrum*) [59] and huaya (*Melicoccus bijugatus*) [25]. The percentage of crystallinity obtained for the starch analyzed in this work was 32 ± 1%. This value is similar to that reported by other authors, as is the case of ramon starch (*Brosimum alicastrum*) with 31.89% and 29.61% [59]. For the starch obtained from the same source, but using another isolation method, the value was found to be 30.56% [62] and for that obtained from huaya (*Melicoccus bijugatus*) around 31.22% [25].

### 3.9. Thermogravimetric Analysis (TGA)

**Figure 7:**
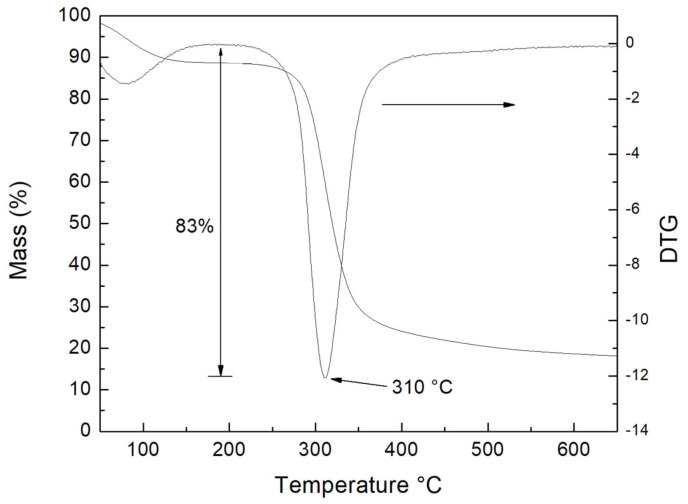
Thermogravimetric analysis of native colok starch.

Thermal analysis results of colok starch are shown in Figure 7, where three stages can be identified. In the first of them, reduction of the mass at 100 °C is notable, a fact that is due to the loss of water [63]. This loss corresponds to 17% of the weight of the analyzed starch. In the second stage, which goes from 225 to 390 °C, the mass continues to decrease, which is related to starch molecules denaturation. Colok starch exhibits a decomposition temperature of 310 °C. At this stage, the mass of colok starch decreases by 83%. In the third stage, starting at 391 °C, the remaining mass is stabilized by the rearrangement of the carbon residues from the polymeric chains of amylose and amylopectin as observed by Tian et al. [64]. In addition, it could be seen that from 150 to 290 °C the starch is stable. This interval could process the starch by extrusion or injection, to convert it into some materials to use in food packaging; in fact, could incorporate some additives to make it more stable as any plasticizer or fill [65,66].

## 4. Conclusions

Physical–chemical, structural, thermal and functional properties of colok native starch were studied. Physicochemical characteristics found in colok starch could be used as a thickener in products that require high viscosity. Balance in nutritional and sensory properties in starch is a substitute for dietary fiber and used to improve properties in foods. Thus, colok starch has been used in cookies with a partial replacement for wheat starch. From a morphological point of view, granules are round and classified as medium in size. Functional properties analyzed, such as solubility, swelling power and water retention capacity, indicate it is feasible to incorporate this starch as a food additive in the preparation of sauces, cookies, bread, noodles and food packages, where these studied properties will improve the quality of the mentioned product. However, research will continue on interactions of starch with other components in a food model.

## Figures and Tables

**Table 1:** Mean ± standard deviation of the proximal composition of native starch from *Talisia floresii Standl* (d.b.).

Sample	Moisture (%)	Ashes (%)	Lipids (%)	Crude Fiber (%)	Proteins (%)	Nitrogen-Free Extract (%)
Native starch	9.49 ± 0.52	1.17 ± 0.05	1.60 ± 1.2	3.62 ± 0.41	ND	93.59 ± 1.01

ND: not detected; d.b. = dry base.

**Table 2:** Mean ± standard deviation of the physicochemical characteristics of *Talisia floresii Standl* native starch.

Parameter	Native Starch
Amylose (%)	33.6 ± 1.15
Amylopectin (%)	66.4 ± 2.05
Amylose/amylopectin ratio	0.5 ± 0.01
Starch yield (% d.b.)	42.1 ± 1.0
Total starch (%)	96.0 ± 0.1
pH	6.44 ± 0.09

d.b. = dry base.

**Table 3:** Color characteristics of *Talisia floresii Standl* native starch.

Parameter	Native Starch
L*	50.38 ± 0.3
a*	9.33 ± 0.03
b*	17.16 ± 0.02
Hue angle	61.45 ± 0.19
Chromaticity C	19.5 ± 0.0

**Table 4:** Mean ± standard deviation of the physicochemical characteristics of *Talisia floresii Standl* native starch.

Starch	T_o_ (°C)	T_p_ (°C)	T_c_ (°C)	ΔH_gel_ (J/g)	PHI (%)
Native starch	81 ± 0.5	85 ± 0.25	90 ± 0.2	17 ± 0.41	4.2

## Data Availability

All data are contained within the article.

## References

[1] Bertoft E. (2017). Understanding starch structure: Recent progress. Agronomy.

[2] Londoño-Restrepo S.M., Rincón-Londoño N., Contreras-Padilla M., Acosta-Osorio A.A., Bello-Pérez L.A., Lucas-Aguirre J.C., Quintero V.D., Pineda-Gómez P., del Real-López A., Rodríguez-García M.E. (2014). Physicochemical, morphological, and rheological characterization of Xanthosoma robustum Lego-like starch. Int. J. Biol. Macromol..

[3] Pérez-Pacheco E., Moo-Huchin V., Estrada-León R., Ortiz-Fernández A., May-Hernández L., Ríos-Soberanis C., Betancur-Ancona D. (2014). Isolation and characterization of starch obtained from *Brosimum alicastrum* Swarts Seeds. Carbohydr. Polym..

[4] Viana E.B.M., Oliveira N.L., Ribeiro J.S., Almeida M.F., Souza C.C.E., Resende J.V., Santos L.S., Veloso C.M. (2022). Development of starch-based bioplastics of green plantain banana (*Musa paradisiaca* L.) modified with heat-moisture treatment (HMT). Food Packag. Shelf Life.

[5] Tarahi M., Shahidi F., Hedayati S. (2022). A Novel starch from bitter vetch (*Vicia ervilia*) seeds: A comparison of its physicochemical, structural, thermal, rheological and pasting properties with conventional starches. Int. J. Food Sci. Technol..

[6] Punia S. (2020). Barley starch: Structure, properties and in vitro digestibility—A review. Int. J. Biol. Macromol..

[7] Li C., Dhital S., Gilbert R.G., Gidley M.J. (2020). High-amylose wheat starch: Structural basis for water absorption and pasting properties. Carbohydr. Polym..

[8] Nordin N., Othman S.H., Rashid S.A., Basha R.K. (2020). Effects of glycerol and thymol on physical, mechanical, and thermal properties of corn starch films. Food Hydrocoll..

[9] Zhan Q., Ye X., Zhang Y., Kong X., Bao J., Corke H., Sui Z. (2020). Starch granule-associated proteins affect the physicochemical properties of rice starch. Food Hydrocoll..

[10] Dzul F.J.T. (2007). La Estacionalidad de la Selva Baja Inundable: Su Análisis Mediante Percepción Remota. Ph.D. Thesis.

[11] Poot-Pool W., Cetzal-Ix W., Basu S., Noguera-Savelli E., Noh-Contreras D. (2018). Urban home gardens: A sustainable conservation model for local plants. Urban Horticulture: Sustainability for the Future.

[12] De Villacorzo P.S.C.M. Documento Técnico Unificado de Aprovechamiento Forestal. http://sinat.semarnat.gob.mx/dgiraDocs/documentos/CUSF/07L702050214.pdf.

[13] Estrada-León R.J., Moo-Huchin V.M., Ríos-Soberanis C.R., Betancur-Ancona D., May-Hernández L.H., Carrillo-Sánchez F.A., Cervantes-Uc J.M., Pérez-Pacheco E. (2016). The effect of isolation method on properties of parota (*Enterolobium cyclocarpum*) starch. Food Hydrocoll..

[14] Betancur D.A., Ancona L.A.C., Guerrero R.I., Camelo Matos G., Ortiz D. (2001). Physicochemical and Functional Characterization of Baby Lima Bean (*Phaseolus lunatus*) Starch. Starch-Stärke.

[15] Okekunle M.O., Adebowale K.O., Olu-Owolabi B.I., Lamprecht A. (2020). Physicochemical, morphological and thermal properties of oxidized starches from Lima bean (*Phaseolus lunatus*). Sci. Afr..

[16] AOAC (1925). Official Methods of Analysis of the Association of Official Analytical Chemists.

[17] Martins da Costa J.C., Lima Miki K.S., da Silva Ramos A., Teixeira-Costa B.E. (2020). Development of biodegradable films based on purple yam starch/chitosan for food application. Heliyon.

[18] Can-Cauich C., Sauri-Duch E., Cuevas-Glory L., Betancur-Ancona D., Ortiz-Vázquez E., Ríos-Soberanis C., Chel-Guerrero L., González-Aguilar G., Moo-Huchin V. (2021). Physicochemical properties and stability of pumpkin seed oil as affected by different extraction methods and species. Int. Food Res. J..

[19] Tejeda L. (1992). The thermal descomposition of carbohydrates. II. The descomposition of fiber. Chem. Biochem..

[20] McGuire R.G. (1992). Reporting of objective color measurements. HortScience.

[21] Musacchi S., Serra S. (2018). Apple fruit quality: Overview on pre-harvest factors. Sci. Hortic..

[22] Ratnayake W.S., Hoover R., Warkentin T. (2002). Pea Starch: Composition, Structure and Properties—A Review. Starch-Stärke.

[23] Morrison W.R., Laignelet B. (1983). An improved colorimetric procedure for determining apparent and total amylose in cereal and other starches. J. Cereal Sci..

[24] Zhao X., Jayarathna S., Turesson H., Fält A.-S., Nestor G., González M.N., Olsson N., Beganovic M., Hofvander P., Andersson R. (2021). Amylose starch with no detectable branching developed through DNA-free CRISPR-Cas9 mediated mutagenesis of two starch branching enzymes in potato. Sci. Rep..

[25] Moo-Huchin V.M., Ac-Chim D.M., Chim-Chi Y.A., Ríos-Soberanis C.R., Ramos G., Yee-Madeira H.T., Ortiz-Fernández A., Estrada-León R.J., Pérez-Pacheco E. (2020). Huaya (*Melicoccus bijugatus*) seed flour as a new source of starch: Physicochemical, morphological, thermal and functional characterization. J. Food Meas. Charact..

[26] Sevenou O., Hill S.E., Farhat I.A., Mitchell J.R. (2002). Organisation of the external region of the starch granule as determined by infrared spectroscopy. Int. J. Biol. Macromol..

[27] Zahib I.R., Md Tahir P., Talib M., Mohamad R., Alias A.H., Lee S.H. (2021). Effects of degree of substitution and irradiation doses on the properties of hydrogel prepared from carboxymethyl-sago starch and polyethylene glycol. Carbohydr. Polym..

[28] Tamimi N., Mohammadi Nafchi A., Hashemi-Moghaddam H., Baghaie H. (2021). The effects of nano-zinc oxide morphology on functional and antibacterial properties of tapioca starch bionanocomposite. Food Sci. Nutr..

[29] Monroy Y., Rivero S., García M.A. (2018). Microstructural and techno-functional properties of cassava starch modified by ultrasound. Ultrason. Sonochem..

[30] Rafiq S.I., Jan K., Singh S., Saxena D.C. (2015). Physicochemical, pasting, rheological, thermal and morphological properties of horse chestnut starch. J. Food Sci. Technol..

[31] Delatte S., Doran L., Blecker C., De Mol G., Roiseux O., Gofflot S., Malumba P. (2019). Effect of pilot-scale steam treatment and endogenous alpha-amylase activity on wheat flour functional properties. J. Cereal Sci..

[32] Torbica A., Belović M., Tomić J. (2019). Novel breads of non-wheat flours. Food Chem..

[33] Da Silva Timm N., Ramos A.H., Ferreira C.D., Biduski B., Eicholz E.D., de Oliveira M. (2020). Effects of drying temperature and genotype on morphology and technological, thermal, and pasting properties of corn starch. Int. J. Biol. Macromol..

[34] Marichelvam M.K., Jawaid M., Asim M. (2019). Corn and Rice Starch-Based Bio-Plastics as Alternative Packaging Materials. Fibers.

[35] Lazzarotto S.R.d.S., Lazzarotto M., Silveira A.C.d., Wendling I., Schnitzler E. (2021). Corn starch incorporated with freeze-concentrated Ilex paraguariensis extracts: A potential nutraceutical product. J. Therm. Anal. Calorim..

[36] Saman W.R., Yuliasih I., Sugiarto M. (2019). Physicochemical characteristics and functional properties of white sweet potato starch. Int. J. Eng. Manag. Res..

[37] Qian J., Kuhn M. (1999). Characterization of Amaranthus cruentus and Chenopodium quinoa Starch. Starch-Stärke.

[38] Lindeboom N., Chang P.R., Tyler R.T. (2004). Analytical, Biochemical and Physicochemical Aspects of Starch Granule Size, with Emphasis on Small Granule Starches: A Review. Starch-Stärke.

[39] Yang Z., Xu X., Singh R., de Campo L., Gilbert E.P., Wu Z., Hemar Y. (2019). Effect of amyloglucosidase hydrolysis on the multi-scale supramolecular structure of corn starch. Carbohydr. Polym..

[40] Jiménez-Hernández J., Meneses-Esparza F., Rosendo-Escobar J., Vivar-Vera M.A., Bello-Pérez L.A., García-Suárez F.J. (2011). Extracción y caracterización del almidón de las semillas de Enterolobium cyclocarpum Extraction and characterization of starch from Enterolobium cyclocarpum seeds. CyTA J. Food.

[41] Mclellan M.R., Lind L.R., Kime R.W. (1995). Hue Angle Determinations and Statistical Analysis for Multiquadrant Hunter L, A, B Data. J. Food Qual..

[42] Biduski B., da Silva W.M.F., Colussi R., Halal S.L.d.M.E., Lim L.-T., Dias Á.R.G., Zavareze E.d.R. (2018). Starch hydrogels: The influence of the amylose content and gelatinization method. Int. J. Biol. Macromol..

[43] Yu M., Liu B., Zhong F., Wan Q., Zhu S., Huang D., Li Y. (2021). Interactions between caffeic acid and corn starch with varying amylose content and their effects on starch digestion. Food Hydrocoll..

[44] Iqbal S., Wu P., Kirk T.V., Chen X.D. (2021). Amylose content modulates maize starch hydrolysis, rheology, and microstructure during simulated gastrointestinal digestion. Food Hydrocoll..

[45] Zhang B., Zhang Q., Wu H., Su C., Ge X., Shen H., Han L., Yu X., Li W. (2021). The influence of repeated versus continuous dry-heating on the performance of wheat starch with different amylose content. LWT.

[46] Kusumayanti H., Handayani N.A., Santosa H. (2015). Swelling Power and Water Solubility of Cassava and Sweet Potatoes Flour. Procedia Environ. Sci..

[47] Ulfa G.M., Putri W.D.R., Widjanarko S.B. (2019). The influence of sodium acetate anhydrous in swelling power, solubility, and water binding capacity of acetylated sweet potato starch. AIP Conf. Proc..

[48] Mbougueng P.D., Tenin D., Scher J., Tchiégang C. (2012). Influence of acetylation on physicochemical, functional and thermal properties of potato and cassava starches. J. Food Eng..

[49] Betancur A.D., Chel G.L. (1997). Acid Hydrolysis and Characterization of *Canavalia ensiformis* Starch. J. Agric. Food Chem..

[50] Kaur A., Singh N., Ezekiel R., Guraya H.S. (2007). Physicochemical, thermal and pasting properties of starches separated from different potato cultivars grown at different locations. Food Chem..

[51] Lin J.-H., Kao W.-T., Tsai Y.-C., Chang Y.-H. (2013). Effect of granular characteristics on pasting properties of starch blends. Carbohydr. Polym..

[52] Pérez-Pacheco E., Canto-Pinto J.C., Moo-Huchin V.M., Estrada-Mota I.A., Estrada-León R.J., Chel-Guerrero L. (2016). Thermoplastic starch (TPS)-cellulosic fibers composites: Mechanical properties and water vapor barrier: A review. Composites from Renewable and Sustainable Materials.

[53] Li C., Gong B. (2020). Insights into chain-length distributions of amylopectin and amylose molecules on the gelatinization property of rice starches. Int. J. Biol. Macromol..

[54] Sudheesh C., Sunooj K.V., George J., Kumar S., Sajeevkumar V.A. (2019). Physico-chemical, morphological, pasting and thermal properties of stem flour and starch isolated from kithul palm (*Caryota urens*) grown in valley of Western Ghats of India. J. Food Meas. Charact..

[55] Uzizerimana F., Dang K., Yang Q., Hossain M.S., Gao S., Bahati P., Mugiraneza N.G., Yang P., Feng B. (2021). Physicochemical properties and in vitro digestibility of tartary buckwheat starch modified by heat moisture treatment: A comparative study. NFS J..

[56] Shivaraju V.K., Vallayil Appukuttan S., Kumar S. (2019). The Influence of Bound Water on the FTIR Characteristics of Starch and Starch Nanocrystals Obtained from Selected Natural Sources. Starch-Stärke.

[57] Engel J.B., Ambrosi A., Tessaro I.C. (2019). Development of biodegradable starch-based foams incorporated with grape stalks for food packaging. Carbohydr. Polym..

[58] Ríos-Soberanis C.R., Collí-Pacheco J.P., Estrada-León R.J., Moo-Huchin V.M., Yee-Madeira H.T., Pérez-Pacheco E. (2021). Biocomposites based on plasticized starch: Thermal, mechanical and morphological characterization. Polym. Bull..

[59] Pech-Cohuo S.C., Hernandez-Colula J., Gonzalez-Canche N.G., Salgado-Transito I., Uribe-Calderon J., Cervantes-Uc J.M., Cuevas-Bernardino J.C., Ayora-Talavera T., Pacheco N. (2021). Starch from Ramon seed (*Brosimum alicastrum*) obtained by two extraction methods. MRS Adv..

[60] Hao H., Li Q., Bao W., Wu Y., Ouyang J. (2018). Relationship between physicochemical characteristics and in vitro digestibility of chestnut (*Castanea mollissima*) starch. Food Hydrocoll..

[61] Pozo C., Rodríguez-Llamazares S., Bouza R., Barral L., Castaño J., Müller N., Restrepo I. (2018). Study of the structural order of native starch granules using combined FTIR and XRD analysis. J. Polym. Res..

[62] Rolando Ríos-Soberanis C., Javier Estrada-León R., Manuel Moo-Huchin V., José Cabrera-Sierra M., Manuel Cervantes-Uc J., Arturo Bello-Pérez L., Pérez-Pacheco E. (2016). Utilization of ramon seeds (*Brosimum alicastrum* swarts) as a new source material for thermoplastic starch production. J. Appl. Polym. Sci..

[63] Liu H., Guo X., Li W., Wang X., Lv M., Peng Q., Wang M. (2015). Changes in physicochemical properties and in vitro digestibility of common buckwheat starch by heat-moisture treatment and annealing. Carbohydr. Polym..

[64] Tian Y., Li Y., Xu X., Jin Z. (2011). Starch retrogradation studied by thermogravimetric analysis (TGA). Carbohydr. Polym..

[65] Aldas M., Pavon C., López-Martínez J., Arrieta M.P. (2020). Pine Resin Derivatives as Sustainable Additives to Improve the Mechanical and Thermal Properties of Injected Moulded Thermoplastic Starch. Appl. Sci..

[66] Ma H., Qin W., Guo B., Li P. (2022). Effect of plant tannin and glycerol on thermoplastic starch: Mechanical, structural, antimicrobial and biodegradable properties. Carbohydr. Polym..

